# 2,3-Dibromo-1,3-bis­(4-chloro­phen­yl)propan-1-one

**DOI:** 10.1107/S1600536810045022

**Published:** 2010-11-13

**Authors:** Hoong-Kun Fun, Ching Kheng Quah, Shobhitha Shetty, Balakrishna Kalluraya

**Affiliations:** a X-ray Crystallography Unit, School of Physics, Universiti Sains Malaysia, 11800 USM, Penang, Malaysia; b Department of Studies in Chemistry, Mangalore University, Mangalagangotri, Mangalore 574 199, India

## Abstract

In the title compound, C_15_H_10_Br_2_Cl_2_O, the terminal benzene rings make a dihedral angle of 31.1 (2)° with each other. In the crystal, mol­ecules are stacked along the *a* axis and consolidated by C—H⋯π inter­actions. Short Cl⋯Cl [3.1140 (17) Å] and Br⋯Cl [3.4565 (13) Å] contacts are observed.

## Related literature

For general background to and the biological activity of chalcones, see: Dimmock *et al.* (1999 ▶); Opletalova & Sedivy (1999 ▶); Nowakowska (2007 ▶). For the preparation of the title compound, see: Rai *et al.* (2008 ▶). For the stability of the temperature controller used in the data collection, see: Cosier & Glazer (1986 ▶). For bond-length data, see: Allen *et al.* (1987 ▶). For a related structure, see: Fun *et al.* (2010 ▶).
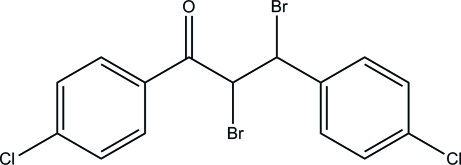


## Experimental

### 

#### Crystal data

C_15_H_10_Br_2_Cl_2_O
*M* *_r_* = 436.95
Orthorhombic, 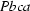

*a* = 5.7599 (3) Å
*b* = 17.1233 (8) Å
*c* = 30.1983 (13) Å
*V* = 2978.4 (2) Å^3^
*Z* = 8
Mo *K*α radiation
μ = 5.79 mm^−1^
*T* = 100 K
0.52 × 0.48 × 0.34 mm

#### Data collection

Bruker SMART APEXII CCD area-detector diffractometer
Absorption correction: multi-scan (*SADABS*; Bruker, 2009 ▶) *T*_min_ = 0.154, *T*_max_ = 0.241
16656 measured reflections
4100 independent reflections
3459 reflections with *I* > 2σ(*I*)
*R*_int_ = 0.039

#### Refinement

*R*[*F* ^2^ > 2σ(*F* ^2^)] = 0.053
*wR*(*F* ^2^) = 0.130
*S* = 1.12
4100 reflections
181 parameters
H-atom parameters constrained
Δρ_max_ = 1.58 e Å^−3^
Δρ_min_ = −0.62 e Å^−3^

### 

Data collection: *APEX2* (Bruker, 2009 ▶); cell refinement: *SAINT* (Bruker, 2009 ▶); data reduction: *SAINT*; program(s) used to solve structure: *SHELXTL* (Sheldrick, 2008 ▶); program(s) used to refine structure: *SHELXTL*; molecular graphics: *SHELXTL*; software used to prepare material for publication: *SHELXTL* and *PLATON* (Spek, 2009 ▶).

## Supplementary Material

Crystal structure: contains datablocks global, I. DOI: 10.1107/S1600536810045022/is2626sup1.cif

Structure factors: contains datablocks I. DOI: 10.1107/S1600536810045022/is2626Isup2.hkl

Additional supplementary materials:  crystallographic information; 3D view; checkCIF report

## Figures and Tables

**Table 1:** Hydrogen-bond geometry (Å, °) *Cg*1 is the centroid of C1–C6 ring.

*D*—H⋯*A*	*D*—H	H⋯*A*	*D*⋯*A*	*D*—H⋯*A*
C11—H11*A*⋯*Cg*1^i^	0.93	2.96	3.638 (5)	131

Symmetry code: (i) 
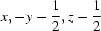
.

**Fig. 1.**
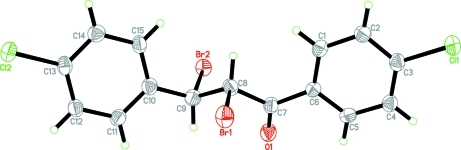
The molecular structure of the title compound showing 50% probability displacement ellipsoids for non-H atoms and the atom-numbering scheme.

**Fig. 2.**
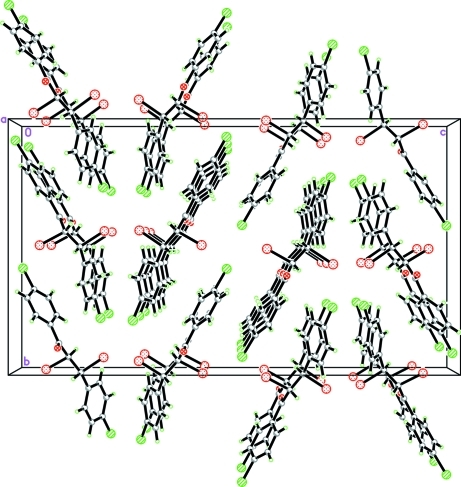
The crystal structure of the title compound, viewed along the a axis.

## supplementary crystallographic information

### Comment

Chalcones are well known for their biological activities (Dimmock *et al.*, 1999). These have been reported as potential antifungal, chemotherapeutic (Opletalova & Sedivy, 1999), anti-infective & anti-inflammatory agents (Nowakowska, 2007). Chalcones are prepared by the condensation of acetophenone with appropriately substituted aromatic aldehydes in ethanol medium employing sodium hydroxide as catalyst. Bromination of these propenones were carried out using bromine in glacial acetic acid medium to give dibromopropanones (Rai *et al.*, 2008). In view of the importance of chalcones, the synthesis and crystal structure of the title compound has been carried out.

In the title molecule, Fig. 1, the benzene rings make a dihedral angle of 31.1 (2) ° with each other. There are short Cl1···Cl1 [symmetry code: -*x*, -*y*, -*z*; distance = 3.1140 (17) Å] and Br1···Cl1 [symmetry code: -1/2-*x*, 1/2+*y*, *z*; distance = 3.4565 (13) Å] contacts which are shorter than the sum of van der Waals radii of chlorine/chlorine and chlorine/bromine atoms. Bond lengths (Allen *et al.*, 1987) and angles are within normal ranges and comparable to a related structure (Fun *et al.*, 2010).

In the crystal packing, Fig. 2, the molecules are stacked down the *a* axis and consolidated by C11—H11A···*Cg*1 (Table 1) interactions, where *Cg*1 is the centroid of the C1–C6 benzene ring. There is no significant hydrogen bond observed in this compound.

### Experimental

1,3-Bis(*p*-chlorophenyl)prop-2-en-1-one (0.01 mol) was dissolved in glacial acetic acid (25 ml) by gentle warming. A solution of bromine in glacial acetic acid [30% (*w*/*v*)] was added to it with constant stirring till the yellow color of the bromine persisted. The reaction mixture was kept aside at room temperature for overnight. Crystals of dibromopropanones separated out were collected by  filtration and washed with ethanol and dried. It was then recrystallized from ethanol. Crystals suitable for X-ray analysis were obtained from ethanol by slow evaporation.

### Refinement

All H atoms were positioned geometrically and refined using a riding model, with C—H = 0.93 or0.98 Å and *U*_iso_(H) = 1.2*U*_eq_(C). The highest residual electron density peak is located at 1.07 Å from Br2 and the deepest hole is located at 1.79 Å from H11A.

### Crystal data

**Table:**

C_15_H_10_Br_2_Cl_2_O	*F*(000) = 1696
*M**_r_* = 436.95	*D*_x_ = 1.949 Mg m^−^^3^
Orthorhombic, *P**b**c**a*	Mo *K*α radiation, λ = 0.71073 Å
Hall symbol: -P 2ac 2ab	Cell parameters from 7210 reflections
*a* = 5.7599 (3) Å	θ = 2.5–29.4°
*b* = 17.1233 (8) Å	µ = 5.79 mm^−^^1^
*c* = 30.1983 (13) Å	*T* = 100 K
*V* = 2978.4 (2) Å^3^	Block, light yellow
*Z* = 8	0.52 × 0.48 × 0.34 mm

### Data collection

**Table:**

Bruker SMART APEXII CCD area-detector diffractometer	4100 independent reflections
Radiation source: fine-focus sealed tube	3459 reflections with *I* > 2σ(*I*)
graphite	*R*_int_ = 0.039
φ and ω scans	θ_max_ = 29.4°, θ_min_ = 2.4°
Absorption correction: multi-scan (*SADABS*; Bruker, 2009)	*h* = −7→6
*T*_min_ = 0.154, *T*_max_ = 0.241	*k* = −23→22
16656 measured reflections	*l* = −41→41

### Refinement

**Table:**

Refinement on *F*^2^	Primary atom site location: structure-invariant direct methods
Least-squares matrix: full	Secondary atom site location: difference Fourier map
*R*[*F*^2^ > 2σ(*F*^2^)] = 0.053	Hydrogen site location: inferred from neighbouring sites
*wR*(*F*^2^) = 0.130	H-atom parameters constrained
*S* = 1.12	*w* = 1/[σ^2^(*F*_o_^2^) + (0.0496*P*)^2^ + 11.6475*P*] where *P* = (*F*_o_^2^ + 2*F*_c_^2^)/3
4100 reflections	(Δ/σ)_max_ = 0.001
181 parameters	Δρ_max_ = 1.58 e Å^−^^3^
0 restraints	Δρ_min_ = −0.62 e Å^−^^3^

### Special details

**Table:**

Experimental. The crystal was placed in the cold stream of an Oxford Cyrosystems Cobra open-flow nitrogen cryostat (Cosier & Glazer, 1986) operating at 100.0 (1) K.
Geometry. All esds (except the esd in the dihedral angle between two l.s. planes) are estimated using the full covariance matrix. The cell esds are taken into account individually in the estimation of esds in distances, angles and torsion angles; correlations between esds in cell parameters are only used when they are defined by crystal symmetry. An approximate (isotropic) treatment of cell esds is used for estimating esds involving l.s. planes.
Refinement. Refinement of F^2^ against ALL reflections. The weighted R-factor wR and goodness of fit S are based on F^2^, conventional R-factors R are based on F, with F set to zero for negative F^2^. The threshold expression of F^2^ > 2sigma(F^2^) is used only for calculating R-factors(gt) etc. and is not relevant to the choice of reflections for refinement. R-factors based on F^2^ are statistically about twice as large as those based on F, and R- factors based on ALL data will be even larger.

### Fractional atomic coordinates and isotropic or equivalent isotropic displacement parameters (Å^2^)

**Table:**

	*x*	*y*	*z*	*U*_iso_*/*U*_eq_	
Br1	−0.01792 (9)	0.49293 (3)	0.070047 (16)	0.03500 (14)	
Br2	0.29597 (8)	0.43618 (3)	0.207223 (14)	0.02966 (13)	
Cl1	−0.0272 (2)	0.08541 (7)	0.01691 (4)	0.0361 (3)	
Cl2	−0.3932 (2)	0.76965 (7)	0.21154 (4)	0.0309 (2)	
O1	0.4810 (6)	0.3947 (2)	0.10860 (12)	0.0343 (8)	
C1	−0.0300 (8)	0.2749 (3)	0.09337 (15)	0.0274 (9)	
H1A	−0.1334	0.3006	0.1120	0.033*	
C2	−0.0933 (8)	0.2049 (3)	0.07360 (15)	0.0279 (9)	
H2A	−0.2362	0.1821	0.0798	0.034*	
C3	0.0597 (8)	0.1696 (3)	0.04456 (15)	0.0281 (9)	
C4	0.2787 (8)	0.2004 (3)	0.03566 (15)	0.0277 (9)	
H4A	0.3794	0.1756	0.0161	0.033*	
C5	0.3415 (8)	0.2684 (3)	0.05661 (14)	0.0267 (9)	
H5A	0.4885	0.2891	0.0517	0.032*	
C6	0.1892 (8)	0.3070 (3)	0.08517 (14)	0.0248 (8)	
C7	0.2737 (8)	0.3804 (3)	0.10644 (15)	0.0275 (9)	
C8	0.0953 (8)	0.4392 (3)	0.12362 (15)	0.0283 (9)	
H8A	−0.0327	0.4120	0.1384	0.034*	
C9	0.2020 (8)	0.4987 (3)	0.15432 (15)	0.0298 (9)	
H9A	0.3422	0.5197	0.1403	0.036*	
C10	0.0499 (8)	0.5659 (3)	0.16830 (15)	0.0292 (9)	
C11	0.1231 (8)	0.6422 (3)	0.16112 (15)	0.0292 (9)	
H11A	0.2644	0.6513	0.1471	0.035*	
C12	−0.0117 (8)	0.7051 (3)	0.17457 (15)	0.0287 (9)	
H12A	0.0393	0.7559	0.1698	0.034*	
C13	−0.2224 (8)	0.6915 (3)	0.19510 (14)	0.0270 (9)	
C14	−0.2990 (8)	0.6163 (3)	0.20252 (15)	0.0297 (9)	
H14A	−0.4406	0.6077	0.2165	0.036*	
C15	−0.1632 (8)	0.5529 (3)	0.18901 (16)	0.0299 (9)	
H15A	−0.2148	0.5021	0.1938	0.036*	

### Atomic displacement parameters (Å^2^)

**Table:**

	*U*^11^	*U*^22^	*U*^33^	*U*^12^	*U*^13^	*U*^23^
Br1	0.0440 (3)	0.0307 (2)	0.0303 (2)	0.0071 (2)	−0.0066 (2)	0.00027 (18)
Br2	0.0316 (2)	0.0294 (2)	0.0280 (2)	0.00218 (17)	−0.00372 (17)	0.00071 (17)
Cl1	0.0440 (6)	0.0255 (5)	0.0389 (6)	−0.0021 (5)	0.0046 (5)	−0.0068 (4)
Cl2	0.0337 (6)	0.0292 (5)	0.0298 (5)	0.0063 (4)	0.0013 (4)	−0.0044 (4)
O1	0.0270 (16)	0.0321 (18)	0.0438 (19)	0.0008 (13)	0.0030 (14)	−0.0076 (14)
C1	0.026 (2)	0.029 (2)	0.028 (2)	0.0040 (17)	0.0015 (17)	−0.0029 (16)
C2	0.026 (2)	0.027 (2)	0.030 (2)	0.0007 (17)	0.0002 (18)	0.0005 (17)
C3	0.036 (2)	0.023 (2)	0.025 (2)	0.0025 (18)	−0.0001 (18)	−0.0002 (16)
C4	0.033 (2)	0.024 (2)	0.027 (2)	0.0034 (18)	0.0016 (18)	−0.0005 (16)
C5	0.026 (2)	0.029 (2)	0.0254 (19)	0.0017 (17)	0.0035 (17)	0.0029 (16)
C6	0.025 (2)	0.026 (2)	0.0229 (18)	0.0006 (16)	0.0006 (16)	−0.0011 (16)
C7	0.031 (2)	0.024 (2)	0.027 (2)	0.0023 (17)	0.0016 (18)	−0.0017 (16)
C8	0.030 (2)	0.022 (2)	0.032 (2)	−0.0006 (17)	0.0004 (18)	0.0007 (17)
C9	0.028 (2)	0.031 (2)	0.031 (2)	−0.0003 (18)	−0.0003 (18)	−0.0021 (18)
C10	0.029 (2)	0.028 (2)	0.030 (2)	−0.0005 (18)	−0.0007 (18)	−0.0037 (17)
C11	0.026 (2)	0.030 (2)	0.031 (2)	0.0004 (18)	0.0027 (18)	−0.0051 (18)
C12	0.033 (2)	0.024 (2)	0.029 (2)	−0.0001 (18)	0.0002 (19)	−0.0018 (17)
C13	0.032 (2)	0.023 (2)	0.0254 (19)	0.0044 (17)	−0.0053 (18)	−0.0014 (16)
C14	0.026 (2)	0.032 (2)	0.032 (2)	0.0006 (18)	0.0003 (18)	−0.0002 (18)
C15	0.031 (2)	0.022 (2)	0.037 (2)	0.0021 (17)	0.0011 (19)	−0.0038 (17)

### Geometric parameters (Å, °)

**Table:**

Br1—C8	1.972 (5)	C7—C8	1.529 (6)
Br2—C9	1.997 (5)	C8—C9	1.508 (6)
Cl1—C3	1.740 (5)	C8—H8A	0.9800
Cl2—C13	1.733 (5)	C9—C10	1.507 (6)
O1—C7	1.221 (6)	C9—H9A	0.9800
C1—C2	1.389 (6)	C10—C11	1.390 (7)
C1—C6	1.399 (6)	C10—C15	1.395 (7)
C1—H1A	0.9300	C11—C12	1.389 (6)
C2—C3	1.382 (6)	C11—H11A	0.9300
C2—H2A	0.9300	C12—C13	1.382 (7)
C3—C4	1.393 (7)	C12—H12A	0.9300
C4—C5	1.374 (6)	C13—C14	1.380 (6)
C4—H4A	0.9300	C14—C15	1.399 (6)
C5—C6	1.397 (6)	C14—H14A	0.9300
C5—H5A	0.9300	C15—H15A	0.9300
C6—C7	1.493 (6)		
			
C2—C1—C6	120.0 (4)	Br1—C8—H8A	110.3
C2—C1—H1A	120.0	C10—C9—C8	116.8 (4)
C6—C1—H1A	120.0	C10—C9—Br2	110.0 (3)
C3—C2—C1	118.9 (4)	C8—C9—Br2	103.9 (3)
C3—C2—H2A	120.6	C10—C9—H9A	108.6
C1—C2—H2A	120.6	C8—C9—H9A	108.6
C2—C3—C4	122.3 (4)	Br2—C9—H9A	108.6
C2—C3—Cl1	118.9 (4)	C11—C10—C15	119.1 (4)
C4—C3—Cl1	118.8 (3)	C11—C10—C9	119.9 (4)
C5—C4—C3	118.1 (4)	C15—C10—C9	121.0 (4)
C5—C4—H4A	121.0	C12—C11—C10	120.9 (4)
C3—C4—H4A	121.0	C12—C11—H11A	119.6
C4—C5—C6	121.3 (4)	C10—C11—H11A	119.6
C4—C5—H5A	119.3	C13—C12—C11	119.5 (4)
C6—C5—H5A	119.3	C13—C12—H12A	120.3
C5—C6—C1	119.4 (4)	C11—C12—H12A	120.3
C5—C6—C7	117.3 (4)	C14—C13—C12	120.7 (4)
C1—C6—C7	123.2 (4)	C14—C13—Cl2	119.5 (4)
O1—C7—C6	120.7 (4)	C12—C13—Cl2	119.8 (4)
O1—C7—C8	120.5 (4)	C13—C14—C15	119.9 (4)
C6—C7—C8	118.8 (4)	C13—C14—H14A	120.1
C9—C8—C7	112.3 (4)	C15—C14—H14A	120.1
C9—C8—Br1	108.9 (3)	C10—C15—C14	119.9 (4)
C7—C8—Br1	104.5 (3)	C10—C15—H15A	120.0
C9—C8—H8A	110.3	C14—C15—H15A	120.0
C7—C8—H8A	110.3		
			
C6—C1—C2—C3	−2.6 (7)	C7—C8—C9—C10	171.9 (4)
C1—C2—C3—C4	2.4 (7)	Br1—C8—C9—C10	56.6 (5)
C1—C2—C3—Cl1	−175.7 (3)	C7—C8—C9—Br2	−66.8 (4)
C2—C3—C4—C5	−0.3 (7)	Br1—C8—C9—Br2	178.0 (2)
Cl1—C3—C4—C5	177.8 (3)	C8—C9—C10—C11	−124.5 (5)
C3—C4—C5—C6	−1.6 (7)	Br2—C9—C10—C11	117.4 (4)
C4—C5—C6—C1	1.4 (7)	C8—C9—C10—C15	56.4 (6)
C4—C5—C6—C7	179.9 (4)	Br2—C9—C10—C15	−61.7 (5)
C2—C1—C6—C5	0.8 (7)	C15—C10—C11—C12	0.5 (7)
C2—C1—C6—C7	−177.6 (4)	C9—C10—C11—C12	−178.6 (4)
C5—C6—C7—O1	−19.2 (6)	C10—C11—C12—C13	−0.5 (7)
C1—C6—C7—O1	159.2 (5)	C11—C12—C13—C14	0.4 (7)
C5—C6—C7—C8	158.0 (4)	C11—C12—C13—Cl2	−179.4 (4)
C1—C6—C7—C8	−23.5 (6)	C12—C13—C14—C15	−0.3 (7)
O1—C7—C8—C9	−17.8 (6)	Cl2—C13—C14—C15	179.5 (4)
C6—C7—C8—C9	164.9 (4)	C11—C10—C15—C14	−0.5 (7)
O1—C7—C8—Br1	100.1 (5)	C9—C10—C15—C14	178.6 (4)
C6—C7—C8—Br1	−77.2 (4)	C13—C14—C15—C10	0.4 (7)

### Hydrogen-bond geometry (Å, °)

**Table:**

Cg1 is the centroid of C1–C6 ring.

**Table:**

*D*—H···*A*	*D*—H	H···*A*	*D*···*A*	*D*—H···*A*
C11—H11A···Cg1^i^	0.93	2.96	3.638 (5)	131

Symmetry codes: (i) *x*, −*y*−1/2, *z*−1/2.
